# Cyclodextrins and Iatrogenic Hearing Loss: New Drugs with Significant Risk

**DOI:** 10.3389/fncel.2017.00355

**Published:** 2017-11-08

**Authors:** Mark A. Crumling, Kelly A. King, R. Keith Duncan

**Affiliations:** ^1^Department of Otolaryngology-Head & Neck Surgery, Kresge Hearing Research Institute, University of Michigan, Ann Arbor, MI, United States; ^2^Audiology Unit, Otolaryngology Branch, National Institute on Deafness and Other Communication Disorders, National Institutes of Health, Bethesda, MD, United States

**Keywords:** ototoxicity, cochlea, outer hair cell, Niemann-Pick disease type C, cholesterol, cyclodextrin, deafness

## Abstract

Cyclodextrins are a family of cyclic oligosaccharides with widespread usage in medicine, industry and basic sciences owing to their ability to solubilize and stabilize guest compounds. In medicine, cyclodextrins primarily act as a complexing vehicle and consequently serve as powerful drug delivery agents. Recently, uncomplexed cyclodextrins have emerged as potent therapeutic compounds in their own right, based on their ability to sequester and mobilize cellular lipids. In particular, 2-hydroxypropyl-β-cyclodextrin (HPβCD) has garnered attention because of its cholesterol chelating properties, which appear to treat a rare neurodegenerative disorder and to promote atherosclerosis regression related to stroke and heart disease. Despite the potential health benefits, use of HPβCD has been linked to significant hearing loss in several species, including humans. Evidence in mice supports a rapid onset of hearing loss that is dose-dependent. Ototoxicity can occur following central or peripheral drug delivery, with either route resulting in the preferential loss of cochlear outer hair cells (OHCs) within hours of dosing. Inner hair cells and spiral ganglion cells are spared at doses that cause ~85% OHC loss; additionally, no other major organ systems appear adversely affected. Evidence from a first-to-human phase 1 clinical trial mirrors animal studies to a large extent, indicating rapid onset and involvement of OHCs. All patients in the trial experienced some permanent hearing loss, although a temporary loss of function can be observed acutely following drug delivery. The long-term impact of HPβCD use as a maintenance drug, and the mechanism(s) of ototoxicity, are unknown. β-cyclodextrins preferentially target membrane cholesterol, but other lipid species and proteins may be directly or indirectly involved. Moreover, as cholesterol is ubiquitous in cell membranes, it remains unclear why OHCs are preferentially susceptible to HPβCD. It is possible that HPβCD acts upon several targets—for example, ion channels, tight junctions (TJ), membrane integrity, and bioenergetics—that collectively increase the sensitivity of OHCs over other cell types.

## Introduction

Ototoxicity in the form of iatrogenic hearing loss arising from various pharmacological treatments has been well-described for more than 1000 years (Schacht and Hawkins, 2006). In these cases, drug treatments, often administered for life-threatening diseases, bring to bear a dilemma balancing the risk to hearing with the desire to remedy disease. Recently, a new class of ototoxic compounds was identified—cyclodextrins (Ward et al., 2010; Crumling et al., 2012; Davidson et al., 2016). Though these compounds have many roles in industrial and medicinal applications as solvents and stabilizers, the risk to hearing only became apparent when highly concentrated doses of cyclodextrin were being evaluated as a treatment for the devastating neurological disorder, Niemann-Pick Disease Type C (NPC). In this review article, we summarize the nature of these compounds, the evidence of cyclodextrin-induced hearing loss in human patients and animal models, and speculate about potential mechanisms that may underlie this ototoxicity.

### Cyclodextrin Types and Structure

**Figure 1:**
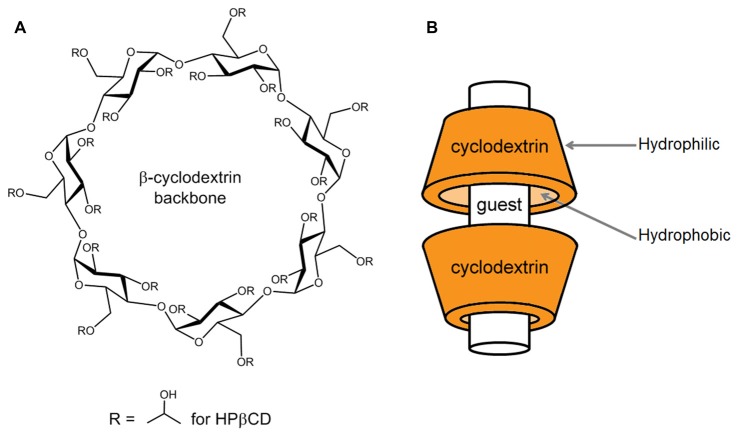
Basic structure of a β-cyclodextrin and mode of complexation with guest compounds. **(A)** The chemical structure of a β-cyclodextrin backbone is illustrated, depicting the seven glucose units and sites of chemical substitution (R). In the native cyclodextrin, a hydrogen occupies each R site. As an example, in the case of 2-hydroxypropyl-β-cyclodextrin (HPβCD), the R sites are occupied to varying numbers by 2-hydroxypropyl groups, as shown. **(B)** Guest compounds can incorporate into the hydrophobic interior of the toroidal cyclodextrin molecule, where they are protected from water. The drawing is modeled after the interaction of HPβCD with cholesterol, where HPβCD dimers can encapsulate one cholesterol molecule (López et al., 2011, 2013).

Cyclodextrins are ring-shaped oligosaccharides formed in nature by the digestion of cellulose by bacteria. They are composed of varying numbers of glucose units held together by α-1, 4 glycosidic bonds. The naturally occurring varieties contain at least six glucose units, with the most common having six, seven, or eight (so called, α-, β-, and γ- cyclodextrins, respectively). Cyclodextrins with more than eight glucose members are less common in nature and less well characterized, and compounds with five glucose units are only synthetic. Bountiful research has been poured into α-, β-, and γ- cyclodextrins and their properties are well characterized. The ring these molecules form (Figure 1A) is often depicted as a cup-shaped toroid (Figure 1B). The outside of the cup is hydrophilic, and the inside is more hydrophobic. Thus, these chemicals are water soluble with the ability to contain hydrophobic guest molecules within them, singly or as dimers (Figure 1B; López et al., 2011, 2013). The resulting increase in solubility and stability of the guest compounds is the predominant basis for the vast medical, industrial and scientific uses of cyclodextrins. Much effort has been expended on improving and tailoring this characteristic by chemical substitution of the hydrogen in the hydroxyl groups, which form the mouths of the toroidal openings, extending from the glucose units. Some common substitutions at these sites are methyl, hydroxylpropyl and sulfobutylether groups. Adding these groups occurs with different efficiencies and results in different sets of impurities along with the intended reaction product. It is chemically difficult to achieve substitution of all possible sites, so a reaction process results in a “degree of substitution”, often expressed as the average number of substituted groups present per molecule or per glucose unit. Different processes produce varied degrees of substitution, and this can have advantages, since both the nature of the substituent group and the degree of substitution influence the performance of the cyclodextrin in ways that can be useful.

### Uses of Cyclodextrins

The ability of cyclodextrins to hold guest compounds has been harnessed in many ways. The food industry has capitalized on this property to entrap various ingredients, masking or preserving flavors in food products. As food additives, the natural α-, β-, and γ- cyclodextrins have the Generally Recognized as Safe (GRAS) label by the U.S. Food and Drug Administration (FDA; Notices 000155, 000074, 000046, respectively), subject to certain percent composition limits. The α- and β-cyclodextrins do not cross the intestinal barrier in significant amounts and are fermented by gut bacteria or excreted whole; γ-cyclodextrins are metabolized by mammalian α-amylases into linear oligosaccharides. Consequently, cyclodextrins in food or otherwise consumed orally do not usually enter the circulation in significant amounts (Frijlink et al., 1990), and thus are generally safe.

Pharmaceutical applications primarily involve the use of cyclodextrins to increase the stability and solubility of drug compounds, but the ability to form inclusion complexes has also been exploited in scavenging applications where injected cyclodextrins can bind to active compounds and end their action on target systems. As constituents of drug formulations, several modified and unmodified cyclodextrins are in the FDA’s Inactive Ingredient Database, suggesting that they are relatively inert up to certain dosages. Safety is greatly increased by hydroxypropyl and sulfobutylether substitution, which has allowed so-modified cyclodextrins to be administered parenterally at high doses to experimental animals with little morbidity and mortality and few obvious side effects (Gould and Scott, 2005). High dosing due to the relative safety of the substituted versions has opened the door to cyclodextrins being used for their own drug effects, rather than just for the actions of guest compounds.

New medical applications have arisen from the insight that uncomplexed cyclodextrins—those with an empty central cavity—can extract and shuttle membrane lipids. Therapeutic applications include treatments for atherosclerosis, Alzheimer’s disease, Parkinson’s disease, infectious disease, and lipid storage disorders (Dass and Jessup, 2000; Graham et al., 2003; Yao et al., 2012; Ottinger et al., 2014; Oliveri and Vecchio, 2016; Zimmer et al., 2016). A recent report generated considerable excitement, showing that 2-hydroxypropyl-β-cyclodextrin (HPβCD) reversed atherosclerosis and limited the formation of new sclerotic plaques in mice, even when the mice were fed a cholesterol-rich diet (Zimmer et al., 2016). The affordability and (reported) safety of HPβCD makes it attractive compared to other treatments for cardiovascular disease, especially for patients who are sensitive to statins or cannot maintain a low-fat, low-cholesterol diet. Similar dosing has been used successfully to treat a mouse model of Alzheimer’s disease (Yao et al., 2012), further increasing the clinical interest in HPβCD. However, the true surge in attention given HPβCD has been driven by its ability to normalize lipid homeostasis and prolong survival in animal models of NPC.

### Cyclodextrins in Treatment of Niemann-Pick Disease Type C

NPC is a rare (1:120,000–1:150,000) autosomal recessive disorder of lipid metabolism characterized by endolysosomal accumulation of unesterified cholesterol (Vanier and Millat, 2003; Vanier, 2010). It is part of a family of metabolic storage disorders, and the hallmark phenotype includes hepatic dysfunction and progressive neurodegeneration. It is panethnic, primarily affects children, and is fatal in all cases.

Biallelic mutations in one of two genes (*NPC1* and *NPC2*) result in NPC, although the two complementation groups appear biochemically indistinguishable and the majority of cases (approximately 95%) occur from DNA-variants in *NPC1* (Carstea et al., 1997; Naureckiene et al., 2000; Ikonen and Hölttä-Vuori, 2004), which, to date, is associated with hundreds of pathogenic mutations. NPC proteins reside in late endosomes/lysosomes, and their exact functions remain unclear (Vanier, 2010); affected cells fail to mobilize cholesterol across cell membranes resulting in excessive and ultimately pathological storage of exogenous, unesterified cholesterol and other lipid moieties in cells and tissues throughout the body (Liscum and Faust, 1987; Liscum et al., 1989). The effect is preferentially severe in neurons and lipid-dense regions of the central nervous system.

The NPC phenotype is complex and heterogeneous. Classical onset occurs in childhood, although presentation can range from the perinatal period to adulthood (Vanier and Millat, 2003), and there is often a diagnostic delay. Early clinical markers tend to involve the hepatic system, however, diagnosis is usually tied to onset of neurological symptoms, such as cerebellar ataxia, dysarthria and cognitive impairment. Vertical supranuclear gaze palsy is considered nearly pathognomic, particularly when coupled with gelastic cataplexy (loss of muscle tone that can be triggered by laughing). Although variable, most patients die in adolescence, 10–15 years after onset of neurological disease (Ory et al., 2017).

Pharmaceutical statins used to treat hypercholesterolemia and dietary cholesterol restriction have not proven effective at preventing or slowing neurological progression in NPC (Patterson et al., 1993; Somers et al., 2001). Miglustat (Zavesca), a small iminosugar that crosses the blood-brain barrier and inhibits an early enzyme in the glycosphingolipid pathway (Patterson et al., 2007), is used for the treatment of Gaucher disease, another disorder of lysosomal storage. Miglustat is an approved therapy for neurological symptoms of NPC in at least 45 countries (Patterson and Walkley, 2017); however its ability to delay neurological progression is modest, and it does not mobilize intracellular cholesterol in NPC. It is currently not approved in the United States for the treatment of NPC, although many patients pursue off-label usage if cost or insurance coverage is not prohibitive.

Recognition of a cyclodextrin derivative as a potential therapeutic intervention for NPC was first reported by Camargo et al. (2001), although the described effect on neurological symptoms was slight and, at the time, cyclodextrin was not considered to be a viable therapy for patients. Renewed attention came when, serendipitously, parallel work from the Dietschy and Walkley labs using HPβCD as an excipient to administer the drug allopregnanolone in a mouse model for NPC showed that HPβCD alone was effective at treating the disease (Davidson et al., 2009; Liu et al., 2009). This confirmed and expanded earlier evidence that HPβCD is efficacious at mobilizing cholesterol in cells (Kilsdonk et al., 1995; Liu et al., 2003). The combination of these discoveries launched an extensive exploration of HPβCD as a potential disease-altering therapy for NPC.

To date, in both feline and mouse models, HPβCD has increased lifespan and ameliorated the neurodegenerative phenotype. In *Npc1*^−/−^ mice, intraperitoneal administration of HPβCD improved liver function, delayed onset and slowed progression of neurological disease while significantly increasing lifespan (Camargo et al., 2001; Davidson et al., 2009). Cats with a single spontaneous missense mutation in *NPC1* develop a phenotype similar to that observed in humans with classical juvenile onset (Lowenthal et al., 1990), and direct intracisternal administration of HPβCD into presymptomatic NPC1 cats prevented onset of cerebellar dysfunction for over a year. In symptomatic animals it slowed neurodegeneration while increasing lifespan. Subcutaneous HPβCD administration was effective at treating peripheral hepatic disease, but required higher doses to treat neurological disease, resulting in pulmonary toxicities (Vite et al., 2015).

It is not clear how HPβCD mechanistically alters the course of NPC disease progression, yet biomarkers show reduced neuronal storage and extended Purkinje cell survival in treated animals. Preclinical studies have confirmed the route of administration impacts efficacy and toxicity, likely related, in part, to HPβCD not crossing the blood-brain barrier at appreciable levels (Pontikis et al., 2013). When HPβCD is delivered directly to the CNS in *NPC1*^−/−^ cats, the effect on survival duration is more than three times as much as when the drug is administered systemically (Vite et al., 2015).

Compelling preclinical data and a unique and innovative partnership established between the scientific and family stakeholder communities (Walkley et al., 2016) have accelerated the move of HPβCD into human phase 1/2a clinical trials, exploring both intravenous (IV) and intrathecal drug delivery using two different preparations (Trappsol Cyclo and VTS-270, respectively). The published results of these early phase studies, focused largely on safety and dosing, show high promise and indicate therapeutic efficacy at treating NPC1 disease in patients (Ory et al., 2017). An ongoing phase 2b/3 clinical trial is nearing completion, and in early 2016 the FDA granted a Breakthrough Therapy designation for a HPβCD preparation (VTS-270), which had previously been given Orphan Drug status. The combination of these designations by the FDA acknowledges the high therapeutic potential to address an unmet need in a rare disease population, and may serve to fast-track HPβCD approval as a therapy for NPC. Preclinical and now, first-in-human data demonstrate it is generally well tolerated, with a single salient toxicity observed thus far across species and now in humans: hearing loss. Despite its ototoxic properties, approval of HPβCD as a therapy for NPC seems likely, and exploration into its use to treat other, more common diseases such as atherosclerosis, is underway (Coisne et al., 2016; Zimmer et al., 2016). Establishing the ototoxic profile and causative mechanisms is critical if HPβCD ototoxicity is to be mitigated or prevented.

## HPβCD Ototoxicity

### Animal Models

Although the toxicology of cyclodextrins has been studied for decades, only in the last several years has their effect on hearing been appreciated. Prior to the discovery of ototoxicity, cyclodextrins, particularly HPβCD, had been administered parentally at doses that were high enough to be ototoxic (reviewed in Frömming and Szejtli, 1994; Gould and Scott, 2005). However, it appears that such doses were given infrequently, and audiological testing was not a priority. Behavioral effects of hearing loss also seem to have gone unnoticed. Only when research on the use of HPβCD to treat NPC in preclinical models prompted routine testing in animals at multi-thousand mg/kg parenteral doses, did the ototoxicity of HPβCD come to light.

### HPβCD Ototoxicity in Cats

The first hint of HPβCD ototoxicity came from NPC research in cats (Ward et al., 2010). Human patients with *NPC1* mutations can have disease-related hearing loss (Pikus, 1991; King et al., 2014), and because of this, the auditory system of NPC cats was being monitored by auditory brainstem response (ABR) recording in experiments on HPβCD as a therapy. NPC cats turned out not to share the auditory phenotype with humans. However, hearing abnormalities were found in animals treated with HPβCD. Elevated ABR thresholds were noted after receiving a single subcutaneous injection of HPβCD at 8000 mg/kg (Ward et al., 2010). Comparable results were found after a single intrathecal injection of 4000 mg/kg brain weight. The elevated thresholds remained unchanged for up to 12 weeks after these single injections. These experiments indicated that single doses capable of altering NPC symptoms were harmful to the auditory system. When subcutaneous injections were given to normal cats weekly at 4000 mg/kg, ABR thresholds were significantly elevated by 4 weeks after the first injection, though single injections at this dose caused no significant threshold shift. This deviation from the behavior with a single subcutaneous injection means that there was a buildup of cyclodextrin or there was an accumulation of effect produced by the cyclodextrin on the auditory system, turning an innocuous dose into one that causes hearing loss. Interestingly, two cats treated at 4000 mg/kg brain weight intrathecally every 2 weeks for 13 weeks showed no response to short-duration, predominantly high frequency, broadband clicks in the ABR testing at the highest level available (125 dB SPL). In a subsequent study (Vite et al., 2015), several groups of NPC cats received HPβCD intracisternally at different concentrations and dosing frequencies. Successful disease treatment was always associated with ototoxicity. The threshold shifts were ~30–50 dB for 30–120 mg HPβCD given intracisternally at 14-day intervals.

### HPβCD Ototoxicity in Mice

From the evaluation of hearing in cats, the permanence of HPβCD induced hearing loss was uncertain and the site of injury was unclear. Moreover, the generalization of the ototoxicity to other mammalian species was unknown. Reports now confirm generalization to mouse (Crumling et al., 2012; Cronin et al., 2015; Patterson et al., 2016). Mice injected with a single subcutaneous dose of 8000 mg/kg HPβCD had ABR thresholds at 4, 16 and 32 kHz that were ~60 dB above those of control animals (Crumling et al., 2012). These elevated thresholds were accompanied by elimination of distortion product otoacoustic emission (DPOAE) responses, indicating outer hair cell (OHC) dysfunction to a degree suggesting that most, if not all, of the ABR deficits were due to OHC pathophysiology. Inspection of organ of Corti whole-mount preparations revealed that the presence of inner hair cells was the same as vehicle-injected controls, but there was a total loss of OHCs over the basal-most ~85% of the sensory epithelium, indicating that the hearing loss produced by HPβCD is permanent under these conditions and confirming the predominant involvement of OHCs.

Surprisingly, while most mice injected at 8000 mg/kg displayed widespread damage to the organ of Corti and physiological losses, the histology and audiometry of some animals were unaffected by the dosing, appearing to be normal (Crumling et al., 2012). There was no middle ground in the ototoxicity, with the effect of HPβCD appearing to be an all-or-none phenomenon. At intermediate doses, the average threshold shifts were reduced but no individual animal displayed intermediate threshold shifts. Instead, the proportion of animals with normal or ~60-dB-elevated ABR thresholds changed to produce an intermediate mean response compared to higher doses (Patterson et al., 2016). This result is similar to that in animals co-treated with an aminoglycoside and a loop diuretic. This combination can produce rapid loss of hair cells, even at doses where the individual drugs do not cause an organ of Corti lesion. In this situation, the loop diuretic allows the aminoglycoside to cross the blood-labyrinth barrier in higher amounts than it achieves when administered alone, with a critical result being high endolymph concentration, allowing the aminoglycoside to enter hair cells via permeation through mechanotransduction channels. In mice, high-dose combinations of kanamycin and bumetanide or furosemide have been seen to eliminate all OHCs in about 80% of animals, while the remaining animals were unaffected by the combination or only had scattered OHC loss (Oesterle et al., 2008; Taylor et al., 2008). Later experiments with kanamycin and a more moderate IV furosemide dose (Jansen et al., 2013; Żak et al., 2016) produced a similar dichotomy. To explain corresponding outcomes in cyclodextrin ototoxicity, we envision a two-stage process, where HPβCD would facilitate its own entry into the cochlea only at a high concentration, perhaps achieved by build-up at blood-labyrinth capillary junctions. Once the concentration threshold is crossed, the flood of HPβCD would inevitably achieve an intracochlear concentration that is deadly to all OHCs that experience it. In this way, small differences in the ability of HPβCD to cross into the cochlea could have big effects on intracochlear concentration and the survival of OHCs.

A threshold for extreme HPβCD entry into the cochlea could be a mechanism of the all or no response to single doses, but that does not mean that no HPβCD enters the cochlea at sub-lethal levels. Similar to cats, a single, low subcutaneous dose in mice can leave no overt lasting physiological or histological defects in the cochlea, but with repeated dosing, detectable deficits can result. In an experiment in mice (Crumling et al., 2012), 4000 mg/kg HPβCD did not produce a threshold shift at 4 kHz, 7–10 days after a single dose. However, with weekly administration at this dose, ABR thresholds at 4 kHz began to elevate about 5 weeks after the start of the HPβCD course. Despite the dosing remaining constant, at about 8 weeks the elevation started to reverse reaching pre-drug threshold levels several weeks later, even while continuing the weekly drug injections. This is reminiscent of the phenomenon in noise-induced hearing loss referred to as “toughening” or “conditioning” (Clark et al., 1987; Sinex et al., 1987; Canlon et al., 1988). It may represent low HPβCD levels inducing homeostatic mechanisms at the hair cell or cochlear level that counteract some facet of HPβCD action, thereby allowing the acoustic sensitivity of the cochlea to return to normal while the animal is still receiving HPβCD.

Since treatment of NPC with HPβCD will inevitably require a combination of systemic and central routes of administration—to treat peripheral and central manifestations of the disease—it was important to determine whether otherwise benign systemic and central doses would act synergistically to cause ototoxic effects (Cronin et al., 2015). Penetration of the brain with HPβCD can be increased compared to systemic delivery by administering the compound into cerebrospinal fluid, as has been done in cats (intrathecal and intracisternal injection; Ward et al., 2010; Vite et al., 2015). This strategy increases benefit for the NPC brain, but fails to treat the disease outside the CNS, where systemic dosing is needed. In mice, HPβCD delivered at 500 mg/kg brain weight into cerebrospinal fluid intracerebroventricularly (ICV), left 4 and 16 kHz ABR thresholds statistically unaffected at 1 week after injection. Likewise, these frequencies were unaffected by a subcutaneous injection at 3000 mg/kg. When the two were combined, however, thresholds increased by 40–50 dB and included widespread OHC loss. Thus, seemingly innocuous doses can become devastating to the ear if administered by different routes close in time. This is further evidence that HPβCD is in the cochlea even when hair cells do not die. A mixture of affected and unaffected animals could still be seen with ICV dosing in this series of experiments, suggesting that the all-or-none mechanism is shared by subcutaneous and ICV delivery routes.

### Ototoxicity of Other Cyclodextrins in Mice

The beneficial effect of HPβCD in NPC (and likewise, the ototoxicity) is thought to arise largely from its cholesterol-binding ability. While HPβCD is good at carrying cholesterol, it is not unique among cyclodextrins in this regard. Various α-, β-, and γ-cyclodextrins have been tested in NPC mice for their ability to normalize disease characteristics and screened for ototoxicity in normal mice (Davidson et al., 2016). The two best cyclodextrins at reversing disease abnormalities were HPβCD and 2-hydroxypropyl-γ-cyclodextrin (HPγCD). After one subcutaneous injection at 8000 mg/kg, all animals tested with HPβCD had ABR threshold shifts of about 60 dB. Interestingly, most animals given an equimolar dose of HPγCD had threshold shifts of about 40 dB, but the ABR thresholds of some animals were unaffected. The production of affected and unaffected animals was similar to that seen with single doses of HPβCD, and this segregation persisted after weekly injections that were carried out until the age of 28 weeks. Thus, with a stable dose level of HPγCD, there did not seem to be an expansion of a cochlear lesion with repeated dosing. These results provide evidence that the all-or-none nature of cyclodextrin-ototoxicity reflects biological interanimal variability and not methodological variance related to the injection. The elevated thresholds of HPβCD treated animals remained stable throughout the course of injections, indicating no recovery at this dose but also no additional injury. Because the ototoxicity of HPγCD under comparable circumstances, with equimolar dosing, was less than with HPβCD, it is attractive to think that HPγCD is a better alternative for treatment of NPC. However, its efficacy in disease treatment was correspondingly less than that of HPβCD.

### Human Ototoxicity

Use of HPβCD to treat human NPC disease was first described in peer-reviewed literature for two children by Matsuo et al. (2013, 2014), who reported no measurable change in hearing via ABR after 2 years of both IV and ICV dosing. No auditory data were shown, and no description of test parameters was provided. Maarup et al. (2015) described outcomes in a 12-year-old boy with NPC1 who was given 27 treatments total, roughly every 2 weeks, of 200 mg intrathecal HPβCD over the course of 1.5 years. Pure-tone audiometry was performed prior to the initial exposure and then again before each dose. A progressive increase in threshold at 8 kHz was reported bilaterally, that was greater in the right ear than the left, which was considered ongoing at the time of publication. No impact was observed at lower test frequencies, and the ABR was reported as stable. A summary of the case-study literature to date on compassionate use of HPβCD, which is primarily a collation of abstracts, is presented by Megías-Vericat et al. (2017); detailed accounts of hearing and ototoxicity are not provided.

Recently, results of the only cohort study to date on the use of HPβCD in patients with NPC were published (Ory et al., 2017). This was an open-label, non-randomized, phase 1/2a dose escalation study assessing safety and efficacy of intrathecal administration of HPβCD. Detailed audiology is reported on 14 patients aged 4–24 years at enrollment with genetically confirmed *NPC1* mutations and neurological manifestations, who were followed for 18 months at the National Institutes of Health Clinical Center, Bethesda, MD, USA (Institutional review board-approved protocol 13-CH-0016). Patients were sequentially assigned, in cohorts of three, to receive starting doses ranging from 50 mg to 900 mg intrathecal HPβCD. Doses were administered monthly and advanced, based on tolerance and safety data from individuals and from higher dose cohorts, up to 1200 mg. Comprehensive audiometry, including pure-tone behavioral assessment (0.25–20 kHz) and measurement of DPOAEs, occurred prior to HPβCD exposure (baseline) and then monthly, before each infusion. Some patients were seen for additional testing during acute phases post exposure. A suprathreshold neurodiagnostic ABR was collected at baseline and then every 6 months. Two patients were unable to provide reliable behavioral responses to pure-tone stimuli due to advanced disease status, and their hearing was monitored via DPOAE and ultimately tone burst (0.5–4 kHz) ABR threshold.

All patients in the trial eventually experienced permanent changes in hearing (Ory et al., 2017). Although hearing loss is a manifestation of NPC disease (Pikus, 1991; King et al., 2014), the hearing loss observed in this trial was time-locked to HPβCD exposure. Notably, pre-existing hearing loss in this population complicates the ototoxic profile of HPβCD. At the 18-month time point, change in hearing was more strongly correlated with hearing at baseline than with mean or median total HPβCD dosages; those patients with better hearing at the outset exhibited greater decline in hearing than those with more pre-existing hearing loss. This analysis was limited to a high frequency pure-tone average (4/6/8 kHz).

All patients had some degree of disease-related pre-existing hearing loss, but only half were considered candidates for hearing aids at the time of enrollment. Following 18 months of monthly HPβCD exposure, all patients were audiometrically considered candidates for hearing aids to support communication, and self-reported outcomes by patients and families choosing to use hearing aids were positive. The ototoxic effect typically began in the highest test frequencies and progressively moved to lower test frequencies; 2–8 kHz were most affected (analysis of ultra-high frequency hearing, above 8 kHz, was not reported). The type of loss was sensorineural and the combination of DPOAE and neurodiagnostic ABR data support a site of lesion that is predominantly OHC (King et al., 2015). Variable susceptibility across patients was observed, suggesting one or more factors potentiate the ototoxic properties of the drug. Two siblings showed particular sensitivity to the ototoxic potential of HPβCD exposure, limiting their total dosage, which suggests genetic involvement.

The onset of the hearing loss in humans appears to be rapid, which aligns with preclinical reports (Cronin et al., 2015; Lichtenhan et al., 2017). Some patients reported auditory symptoms (e.g., tinnitus, aural fullness) within hours of the infusion, and loss of OAE and changes in pure-tone thresholds were documented hours after administration. An unanticipated observation was a temporary component to the hearing loss in some cases. Serial monitoring of a subset of patients receiving doses of 600 mg and higher revealed cases in which OAEs were lost and pure-tone thresholds were elevated, followed by a partial or full recovery within a 3–5 week timeframe, prior to subsequent dosing (King et al., 2015, 2016). A similar temporary depression of auditory function has been reported in mice receiving repeated HPβCD exposure (Crumling et al., 2012). The mechanism(s) behind this remains unclear. Objective changes in auditory function often correlated with subjective reports from patients, who noted recovery in their auditory symptoms in the week or two following their infusion. Importantly, not all patients experienced temporary changes in hearing; some patients had no acute change in hearing following a given dose, and others had acute and permanent change. One patient, the youngest and smallest in the cohort, experienced significant and permanent decline in hearing following the initial exposure to 900 mg HPβCD (King et al., 2016).

Data presented by Ory et al. (2017) represent the first cohort of patients systematically evaluated for intrathecal HPβCD safety and efficacy, including comprehensive audiology. The results are promising, as treated patients showed evidence for slowed disease progression. The ototoxicity after 18 months of dosing was considered an expected and acceptable adverse event, when placed in the context of a devastating disease. Treatment of NPC with HPβCD, however, will be a maintenance therapy and it remains unclear what the ototoxic profile will be after years of exposure. ABR threshold data to broadband click stimuli from cats treated intrathecally at therapeutic levels showed profound hearing loss (Vite et al., 2015); although this short-duration click does not effectively probe lower frequency regions of the cochlea, it is possible that chronic exposure may cause widespread cochlear damage. It is also unclear what affect age has on the risk for ototoxicity, but it can be predicted that, should HPβCD be approved as a therapy for NPC, very young patients, newly diagnosed through advances in whole exome sequencing, may be candidates for treatment. The risk for functionally significant hearing loss before development of speech and language should be weighed carefully with potential benefits of early-life treatment of the disease.

## Cyclodextrin Toxicology

Given their widespread use as chemical excipients in drug formulations, as food additives, and as chemical stabilizers in a plethora of industrial products, the toxicological profile of cyclodextrins has been extensively studied and most varieties exhibit low toxicity at low concentrations. Even so, there can be considerable deleterious toxicological effects including death depending on formulation, concentration, and route of delivery. Table 1 shows the LD_50_ data for rats given oral or IV administrations of unmodified cyclodextrins (reviewed by Del Valle, 2004). The greater safety profile for oral delivery likely reflects metabolism by bacteria in upper and lower intestinal tracts and reduced drug absorption compared to IV delivery. Toxicity from IV administration is largely a result of kidney damage, where filtration causes the formation of microcrystals composed of cyclodextrin and possibly deposits of cholesterol, resulting in nephrosis (Frank et al., 1976). Renal damage can occur with modified cyclodextrins as well, depending on concentration, since excretion in urine is the major means of drug elimination. Nephrotoxicity is of particular importance given the link between kidney and inner ear pathology from a variety of ototoxins.

**Table 1:** Toxicology of naturally occurring cyclodextrins in the rat (compiled from Del Valle, 2004).

Cyclodextrin type	Oral LD_50_	IV LD_50_
α-cyclodextrin	>10,000 mg/kg	500–750 mg/kg
β-cyclodextrin	>5000 mg/kg	450–790 mg/kg
γ-cyclodextrin	≫8000 mg/kg	4000 mg/kg

The derivation of modified cyclodextrins has been and continues to be an exciting area of medicinal chemistry research, where substitutions of reactive moieties for various functional groups can range from simple modifications that increase solubility and safety (e.g., methylated, hydroxypropylated and sulfobutylated cyclodextrins) to supramolecular scaffolds and nanoparticles used in drug delivery (Stella and Rajewski, 1997; Varan et al., 2017). Derivatives of β-cyclodextrins, and specifically HPβCD, are widely studied and safety profiles have been extensively reviewed (for example see Gould and Scott, 2005). In an acute study in monkey, a single IV dose of 10,000 mg/kg HPβCD produced no mortality or observed toxicological effects, except hematuria in several animals (Brewster et al., 1990). In a study by AstraZeneca, continuous IV infusion in rat for 7 days reaching an effective dose of 2400 mg/kg/day of HPβCD lowered serum cholesterol and caused minor injury to the kidney (Gould and Scott, 2005). Mild histopathology, with no or limited physiological effects, from this and other short-term studies was generally reversible after cessation of drug treatment. Similarly, examination of several organ systems, including kidney, after 8000 mg/kg subcutaneous HPβCD in mice revealed only minor histopathological effects 3 days post-injection (e.g., edema, vacuolization), even while demonstrating hearing loss and severe OHC loss in those same animals (Cronin et al., 2015). What then underlies HPβCD-induced ototoxicity, and specifically OHC death, for doses that are seemingly benign to other organs and systems?

### Can Indirect Mechanisms Lead to Ototoxicity?

In considering the potential cause(s) of HPβCD-induced ototoxicity, we must first address whether HPβCD ototoxicity is due to the cyclodextrin itself or some indirect effect of HPβCD treatment. These indirect effects could arise, for example, from impurities in the drug formulation or transport of toxic guest compounds “hitching a ride” to the ear during drug delivery. The main by-products of HPβCD synthesis are unreacted β-cyclodextrin and propylene glycol. The amount of impurities increases with the degree of substitution, as greater amounts of NaOH and propylene oxide are required for the reaction (Malanga et al., 2016). A variety of commercial HPβCD products have been examined and proven ototoxic, including formulations from Sigma-Aldrich (C0926, H107; Ward et al., 2010; Crumling et al., 2012; Cronin et al., 2015), Roche (Kleptose HPB; Davidson et al., 2016), and a highly characterized and purified form of Kleptose HPB used in the phase 2/3 clinical trials (VTS-270; Davidson et al., 2016; Ory et al., 2017; Yergey et al., 2017). Moreover, recent reports show little effect of degree of substitution on cell toxicity (Malanga et al., 2016). Since propylene glycol is a presumed ototoxin, it is possible that this contaminant is responsible for the ototoxicity after HPβCD treatment. The toxicity of propylene glycol has been reported for highly concentrated drug applications to the round window or via infusions into the middle ear (Vernon et al., 1978; Morizono et al., 1980), but the sensory epithelium appears well-preserved, with damage primarily occurring in the middle ear and ossification developing in scala tympani and scala vestibuli. Therefore, otopathology due to propylene glycol is quite distinct from that due to HPβCD treatment. Furthermore, the concentrations of this impurity are likely below normal dose limits. Propylene glycol is a common solvent for pharmaceuticals and dose limits for IV administration are on the order of 69 g per day for humans (Lim et al., 2014). Pharmacopoeial specifications (EP/USP) for HPβCD formulations limit propylene glycol contamination to a maximum of 2.5%. Highly purified, commercial formulations of HPβCD such as Kleptose HPB contain less than 0.2% propylene glycol (Machielse and Darling, 2017). If this formulation was given to an average human at 8000 mg/kg, the propylene glycol contamination would be less than 1 g, far below the dose limit for humans. Yet, Kleptose HPB (aka VTS-270) is ototoxic in mice and humans (Davidson et al., 2016; Ory et al., 2017). Therefore, HPβCD ototoxicity does not appear to be related to impurities in drug formulation.

Another possibility for indirect ototoxicity is that HPβCD complexes with endogenous compounds prior to entering the inner ear, carrying potentially damaging substances into the cochlea. When HPβCD is injected systemically, it passes through various tissue and vascular compartments, enabling exchange with myriad sterols, phospholipids and proteins. Cholesterol—the most likely endogenous guest compound—is extracted from plasma membranes by cyclodextrin with two distinct kinetic rates on the order of 10 s and 10 min, likely reflecting exchange from different membrane compartments (Rouquette-Jazdanian et al., 2006). Once in the bloodstream, extraction of cholesterol by cyclodextrins from lipoprotein complexes is expected to occur on similar timescales because the exchange of cholesterol between lipoproteins occurs with a t_1/2_ of 4–45 min (Lund-Katz et al., 1982). Even so, although HPβCD is excreted intact in urine (Stella and He, 2008), the amount of cholesterol in the urine was unchanged in mice—wildtype and NPC mutants—after a single subcutaneous dose of 4000 mg/kg HPβCD (Taylor et al., 2012). It is likely that guest compounds, including cholesterol, are mobilized between local cellular and tissue compartments rather than redistributed from one region to another (i.e., from distal compartments to the inner ear). Therefore, it is unlikely that transport of extracochlear, endogenous, toxic guest compounds is responsible for HPβCD-induced ototoxicity.

### Cyclodextrin Cytotoxicity May Involve Membrane Perturbation and Caspase-Dependent Cell Death

Similar to the *in vivo* toxicology of cyclodextrins, *in vitro* cytotoxicity arising from cyclodextrin exposure depends on formulation and concentration. Cell viability after cyclodextrin treatment has been reported for a variety of cultured lines of epithelial cells, including Calu-3 cells (human airway, Matilainen et al., 2008) and Caco-2 cells (human colorectal adenocarcinoma, Kiss et al., 2010). In both examples, HPβCD was less toxic than other forms, showing good viability (80% or better) when used at concentrations of 200 mM for up to 4 h, whereas methylated forms of β-cyclodextrin caused substantial cell death at 10–50 mM. The difference in cytotoxicity among the various derivatives was tightly associated with two factors: (1) their degree of cholesterol solubilization; and (2) the number and position of methyl groups on methylated derivatives (Kiss et al., 2010). Non-methylated β-cyclodextrins, like HPβCD and sulfobutylether-β-cyclodextrin, show lower cholesterol affinity but also lower cytotoxicity (Kiss et al., 2010; Wang et al., 2011).

Reported mechanisms of cytotoxicity generally involve cholesterol depletion from the plasma membrane or intracellular compartments, but the link between cholesterol depletion and cell death remains unclear. Membrane damage, quantified by the release of cytoplasmic enzymes, is a common observation (Boulmedarat et al., 2005; Wang et al., 2011; Hinzey et al., 2012), presumably due to cholesterol extraction and membrane perturbation. Both apoptotic and necrotic cell death pathways appear to be involved, but the balance of these effects is dependent on the type of cyclodextrin as well as the cell type being examined (Wang et al., 2011; Onodera et al., 2013). In some instances, cyclodextrin-induced apoptosis follows activation of executioner caspase-3 as well as a loss of mitochondrial transmembrane potential and cytochrome release (CytC) (Onodera et al., 2013), but it remains unclear whether this occurs through drug effects on the plasma membrane leading to activation of apoptosis inducible factors or direct effects of cyclodextrin on intracellular organelles. In all cases, these effects are associated with methylated β-cyclodextrins. HPβCD concentrations may have to exceed 200 mM to cause similar effects in these cell lines. Why then do OHCs appear to be sensitive to HPβCD concentrations as low as 10 mM (Takahashi et al., 2016)?

### Potential Mechanisms of HPβCD Ototoxicity

**Figure 2:**
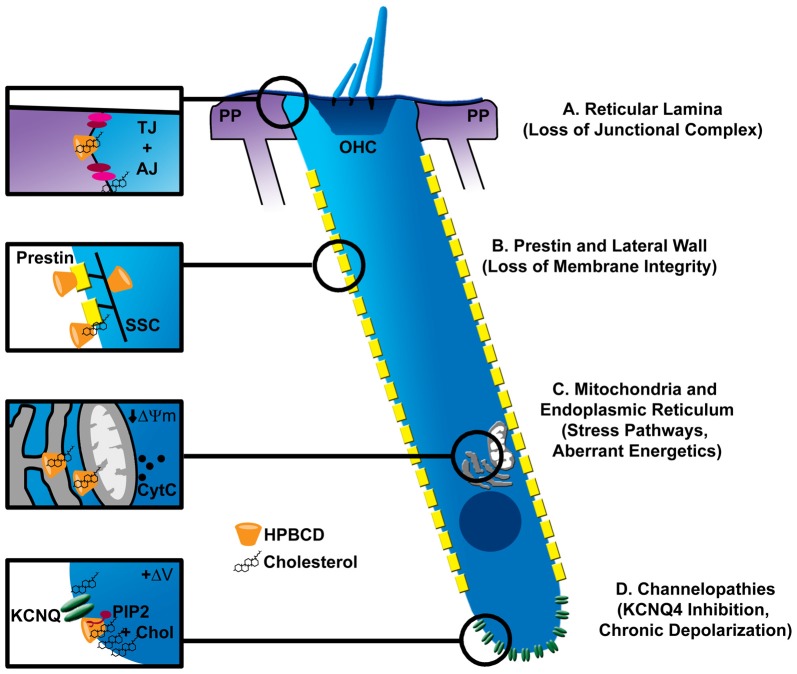
Possible sites of pathologic interaction of cyclodextrins with outer hair cells (OHCs). **(A)** Cyclodextrins disrupt cell-cell junctional complexes (TJ, tight junctions; AJ, adherens junctions), which could breach the reticular lamina of the organ of Corti, allowing the high-potassium endolymph to bathe OHCs, leading to their excitotoxic death by prolonged depolarization. **(B)** The trilaminate structure of the OHC lateral wall is composed of prestin-rich plasma membrane connected to subsurface cisterane (SSC) by a cortical lattice. Removal of membrane cholesterol by cyclodextrins alters OHC membrane fluidity, stiffness and prestin-based motility. Since cholesterol level in the lateral wall appears low, it is unclear if cyclodextrin modifies these properties by interacting with plasma membrane lipids, membrane resident proteins like prestin, or the SSC. Nonetheless, loss of membrane integrity may be one mechanism of cyclodextrin-induced ototoxicity. **(C)** Since cyclodextrins can be endocytosed, they can potentially affect the membranes of intracellular organelles. Mitochondria and the endoplasmic reticulum (ER) represent common targets of other ototoxins. Specifically, cyclodextrins have been shown to reduce the mitochondrial membrane potential (ΔΨm) and cause cytochrome release (CytC). Altered function in either organelle could contribute to OHC demise. **(D)** Cholesterol is non-uniformly distributed in the OHC plasma membrane, being enriched at the apical and basal poles of the cell. High concentrations at the base overlap with the expression of KCNQ4-type potassium channels, which also require the phospholipid PIP2 for proper function. Disruption of a channel-PIP2-cholesterol complex by cyclodextrin could cause loss of KCNQ4 current, chronic depolarization and ultimately excitotoxic death.

Based on the actions of β-cyclodextrins in other systems, these compounds could target at least four aspects of OHC physiology, as depicted in Figure 2. These targets include intracellular organelles that govern a variety of cell stress pathways, membrane resident proteins, and cell-cell junctions that regulate ion flux between fluid compartments surrounding the OHCs. Oxidative stress is a major effector of hair cell death in acquired forms of hearing loss (for review see Dinh et al., 2015; Jiang et al., 2017). Cyclodextrin-induced cytotoxicity in other systems raises the possibility that mitochondrial dysfunction and oxidative stress form a common link with this new ototoxin as well. Several reports suggest that cyclodextrins can enter cells through endocytosis (Rosenbaum et al., 2010; Fenyvesi et al., 2014), deplete cholesterol in mitochondrial membranes, and alter mitochondrial bioenergetics (Ziolkowski et al., 2010; Figure 2C). Cyclodextrins can also disrupt ganglioside-rich domains at the mitochondria-associated endoplasmic reticulum (ER) membranes, linking cyclodextrin treatment to several stress pathways that are also common to other forms of hearing loss (Sano et al., 2009). Though ubiquitous in cell membranes, cholesterol is heterogeneously distributed in mammalian cells; the plasma membrane accounts for 60%–90% of cellular cholesterol and the ER about 1% (Das et al., 2014). Cholesterol homeostasis, one of the mostly tightly controlled processes in mammalian cells, is intimately related to synthesis in the ER and transport between other organelles and the plasma membrane. It is possible that high concentrations of cyclodextrin perturb this balance and overwhelm homeostatic processes to negatively impact vesicular transport between organelles and a large array of cellular processes. It is important to note that most studies linking cholesterol distribution/content and cell function utilize methylated cyclodextrins, so it remains to be seen whether these observations can be extended to HPβCD. Also, if oxidative stress and perturbation of general cell function are the major effectors of HPβCD ototoxicity, it is difficult to explain the rapid nature of the injury and the specificity of OHC death without nephrotoxicity. Further examination of cell death pathways initiated by inner ear exposure to HPβCD is required.

Unique features of OHCs may help explain their sensitivity to HPβCD. Here, it is important to consider the non-homogeneous distribution of cholesterol in the OHC membrane. Cholesterol markers, filipin and theonellamide, intensely label apical and basal OHC regions with little or no staining along the lateral wall (Nguyen and Brownell, 1998; Takahashi et al., 2016). Interestingly, regions of low and high cholesterol mirror the non-homogeneous distribution of key OHC membrane-resident proteins, prestin and KCNQ4, respectively (Mustapha et al., 2009). One of the more obvious features unique to OHCs is related to their function in cochlear amplification via the abundant membrane motor protein prestin and the complex trilaminate structure of the OHC lateral wall (Figure 2B). Indeed, prestin function is exquisitely sensitive to cyclodextrin treatment, such that the voltage-sensitivity of prestin is shifted to more depolarized or hyperpolarized potentials depending on whether cholesterol is depleted or enriched in the OHC membrane (Rajagopalan et al., 2007; Takahashi et al., 2016). Moreover, cholesterol depletion, under certain conditions, alters the fluidity of the OHC lateral membrane (Organ and Raphael, 2009; Yamashita et al., 2015), and recent data suggest involvement of the trilaminate structure of the lateral wall, possibly including the intricate subsurface cistern and its cytoskeletal connections to the plasma membrane (Yamashita et al., 2015). These data suggest potential impacts on membrane integrity, but the exact site of action remains unclear. Interestingly, OHCs from prestin knockout mice are partially protected from HPβCD ototoxicity (Takahashi et al., 2016), suggesting a link between HPβCD and prestin/membrane function in ototoxicity. However, the mechanism of protection in the knockout mice remains unclear. The preservation of OHCs in the knockout animals could reflect the large-scale redistribution of cholesterol throughout the OHC membrane, which would undoubtedly change how HPβCD interacts with the OHCs. Moreover, only a portion of the mutant OHCs were protected from HPβCD, though prestin was absent from both resistant and susceptible OHCs alike. More study is required. It would be interesting to determine whether knock-in of mutated, dysfunctional prestin preserves the normal distribution of lipids in the OHC lateral wall and then renders sensitivity or resistance to HPβCD. It will also be important to determine the pharmacokinetics of HPβCD accumulation in the inner ear fluid spaces to determine whether drug concentration in perilymph reaches levels required to catastrophically damage the OHC membrane (~10 mM).

Other membrane resident proteins, such as ion channels, could be affected by cyclodextrin as well. In chick hair cells, methyl-β-cyclodextrin depleted membrane cholesterol, disrupted membrane microdomains, and altered major ion channel conductances (Purcell et al., 2011). All major ion channel classes are sensitive to perturbations in membrane cholesterol (Levitan et al., 2010), but the effects are heterogeneous and highly context dependent. Membrane perturbation can alter channel density, electrical activity and localization in specific membrane compartments (Levitan et al., 2010; Purcell et al., 2011; Mercer et al., 2012). It is important to emphasize that cholesterol can inhibit or activate the same channel type in different tissues. The potassium channel, KCNQ4, is the dominant potassium conductance in OHCs. Mutations in KCNQ4 are associated with DFNA2, a nonsyndromic progressive form of sensorineural hearing loss characterized by loss of OHCs (Nie, 2008). Expression of KCNQ4 at the basal pole of the OHC overlaps in distribution with cholesterol and other lipids (Santi et al., 1994; Nguyen and Brownell, 1998; Figure 2D). Additionally, KCNQ channel activation is modulated by cholesterol (Chun et al., 2010), possibly through the coordinated perturbation of PIP2, which is required for KCNQ channel gating (Suh and Hille, 2008). A complete understanding of HPβCD effects on OHC physiology is required to fully appreciate the potential mechanisms underlying ototoxicity from this drug.

Finally, it should be noted that HPβCD effects on other structures within the inner ear could indirectly affect OHC health or modulate their susceptibility to direct influence of the drug. One possibility is altered ion homeostasis within the cochlear duct. Direct application of HPβCD into the guinea pig cochlea does not seem to impact endocochlear potential (Lichtenhan et al., 2017) and histopathological effects on the mouse lateral wall appear absent (Crumling et al., 2012; Cronin et al., 2015). However, in many epithelial systems, cyclodextrins effectively disrupt tight-junction barriers (Francis et al., 1999; Lynch et al., 2007), leading to one of their major uses in drug delivery (epidermal patches). Human mutations associated with the integrity of a partition between cochlear fluid compartments—the reticular lamina—are associated with deafness, largely due to OHC loss (Ben-Yosef et al., 2003; Kamitani et al., 2015). Presumably, disruption of the reticular lamina would cause potassium flux into the fluid surrounding the basal pole of the OHCs, resulting in chronic depolarization and possibly cell death (Zenner, 1986; Zenner et al., 1994; Figure 2A).

Each of the potential mechanisms discussed above underscores the unique susceptibility of OHCs to various insults. HPβCD entry into the cochlear duct could induce one or more events that lead to the selective injury of OHCs. More investigation is warranted to determine whether any of these potential mechanisms are at play. Only then can proper interventions be developed.

## Summary and Future Directions

Cyclodextrin use is expanding in medicine, far beyond use as pharmacological excipients. In NPC, HPβCD is the only viable treatment option currently under consideration. In other lipid-related diseases where alternative treatment options exist, HPβCD still potentially offers greater affordability, increased safety, and uniquely attractive properties. To harness the power of this versatile drug, it is imperative to address iatrogenic hearing loss as the major factor limiting further use.

Basic pharmacokinetic and pharmacodynamic studies are needed to understand the relationship between dose, route of administration, accumulation in the ear, transient and permanent hearing loss, and long-term otopathology. These studies could be accomplished by pairing drug injection, auditory tests and pathology with mass spectrometry, which could be used to detect HPβCD in tissue or fluid samples (Jiang et al., 2014), or with radiolabeled HPβCD, which could be used to probe uptake in cellular compartments (Pontikis et al., 2013). These studies could resolve how peak drug concentration and accumulated exposure relate to pathology, and they could define appropriate drug concentrations and exposure durations in *in vitro* models of OHC response to cyclodextrin. Of primary importance to current clinical trials is a greater understanding of the risk from long-term exposure to repeated doses. With long-term use and with combined systemic and central drug administration, otopathology may extend beyond OHC loss to eventually include damage to other cochlear structures including inner hair cells and auditory neurons. Moreover, future studies should examine whether there is risk to hearing from cyclodextrin-based drug formulations, where dissociation of guest compounds might free the excipient to accumulate in the ear and cause injury. Once we know more about the concentrations of HPβCD that reach and reside in the ear over time, we can more fully examine the physiological response of the cochlea during and following peak exposures and build suitable *in vitro* models for examining OHC physiology in response to clinically relevant concentrations of HPβCD.

Along with studies linking pharmacokinetics to injury, we need greater mechanistic insight into the means of HPβCD uptake into the cochlea, the molecular changes that result from HPβCD exposure in the cochlea, and the cell-death pathways that are involved in OHC loss. In this review article, we hypothesized that HPβCD transiently disrupts the blood-labyrinth barrier, facilitating entry into the cochlear duct. Effects on this barrier should be examined further, including examination of changes to endocochlear potential, the morphology of vascular beds, and paracellular extravasation of the drug into the cochlea. The studies with radiolabeled HPβCD could also be informative here to determine whether there is cellular uptake and whether the drug is targeted to specific cellular compartments. Basic morphological studies are needed to derive the primary mechanisms of cell death (e.g., apoptosis, necrosis, or a combination of these) and could reveal key structural changes (e.g., loss of membrane integrity, apoptotic or necrotic nuclei) or molecular changes (e.g., caspase-dependent/independent apoptosis) that lead to OHC demise.

Figure 2 illustrates several potential targets that could link cyclodextrin effects to cholesterol perturbation, cellular dysfunction and ultimately cell death. One of these targets includes the junctional complexes between sensory and non-sensory cells that separate fluid compartments in the cochlea. Morphological studies could help resolve whether these junctional complexes along the reticular lamina are disrupted after HPβCD administration. Other potential targets include membrane processes essential for OHC function, like prestin-mediated motility, lateral wall stiffness and fluidity, and ion channel function. It remains unclear whether systemic administration of HPβCD alters OHC physiology. With a greater understanding of HPβCD pharmacokinetics, OHC function could be examined *in vitro* using drug applications that mirror *in vivo* drug concentrations; alternatively, OHC function could be assessed *in vitro* at specified points after systemic drug injection. Finally, from the evidence that cyclodextrins can negatively impact mitochondrial function and the link between mitochondrial dysfunction, oxidative stress and hearing loss from other ototoxins, future studies should examine potential relationships between HPβCD treatment and OHC oxidative stress.

Preventative approaches, namely adjuvants that might prevent HPβCD ototoxicity, must be pursued in parallel with mechanistic studies since there is now overwhelming evidence that human patient hearing is at risk. Moreover, the potential expansion of NPC clinical trials to young presymptomatic patients raises the specter of causing hearing loss during critical phases of cognitive development in otherwise phenotypically normal patients. Such preventative measures might focus on antioxidants that are currently in clinical trial for other forms of acquired hearing loss (e.g., NCT02903355, NCT01727492), compounds that would modulate OHC excitability and membrane function, and compounds that might modulate uptake and egress of HPβCD into and from the cochlear duct. The results from both mechanistic and therapeutic studies will undoubtedly shape our understanding of cochlear physiology and OHC structure-function specifically. A conundrum has been raised in this review article: HPβCD operates primarily through cholesterol manipulation and cholesterol is a ubiquitous component of cell membranes, yet OHCs show exceptional sensitivity to HPβCD exposure, more so than apparently any other cell in the body. Certainly, we stand to gain major insights into OHC physiology and OHC membrane function by further pursuing the mechanisms of cyclodextrin ototoxicity.

## Author Contributions

MAC, KAK and RKD contributed to conception and design, review and interpretation of literature and drafting and revising the manuscript.

## Conflict of Interest Statement

The authors declare that the research was conducted in the absence of any commercial or financial relationships that could be construed as a potential conflict of interest.
